# Forecasting the Probability That Each Surgical Case Will Either Be Ambulatory or the Patient Will Remain in the Hospital Overnight Versus Having a Length of Stay of Two or More Days

**DOI:** 10.7759/cureus.10847

**Published:** 2020-10-08

**Authors:** Franklin Dexter, Richard H Epstein, Pengyi Shi

**Affiliations:** 1 Anesthesiology, University of Iowa, Iowa City, USA; 2 Anesthesiology, University of Miami Miller School of Medicine, Miami, USA; 3 Operations Research, Purdue Univeristy, West Lafayette, USA

**Keywords:** length of stay, operating room, hospitalization, censuses, ambulatory care

## Abstract

When the hospital census is high, perioperative medical directors or operating room (OR) managers sometimes need to review with surgical departments as to which surgical cases scheduled to be performed within the next three days may need to be postponed. Although distributions of hospital length of stay (LOS) are highly skewed, a surprisingly effective summary measure is the percentage of patients previously undergoing the same category of procedure as that scheduled whose LOS was zero or one day. We evaluated how to forecast each hospital's percentage of cases with LOS of <2 days, segmented by category of surgical procedure. The large teaching hospital studied included several inpatient adult surgical suites, an ambulatory surgery center, and a pediatric surgical suite.

We included 98,540 cases in a training dataset to predict 24,338 cases in a test dataset. For each category of procedure, we calculated the cumulative count of cases among quarters, from the most recent quarter, second most recent quarter, and so forth up to the quarter resulting in at least 800 cases. If every quarter combined had fewer than 800 cases for a given category of procedure, we included all cases for that category. For each combination of category and quarter, we used the cumulative counts of cases and cases with LOS of <2 days, excluding the current quarter. Then, for each category of procedure, and for each of the preceding quarters included for the category, we used the cumulative counts to calculate the asymptotic standard error (SE) for the proportion of cases with LOS of <2 days. If all preceding quarters combined provided a sample size such that the estimated SE for the proportion exceeded 1.25%, we included all preceding quarters. The observed absolute percentage error was 0.76% (SE: 0.12%). This error was nearly 100-fold smaller than the percentage of cases to which it would be used (i.e., 0.76% versus 73.1% with LOS of <2 days). The principal weakness of the forecasting methodology was a small bias caused by a progressive reduction in the overall LOS over time. However, this bias is unlikely to be important for predicting cases’ LOS when the hospital census is high. When performing these time series calculations quarterly, a reasonable approach is to perform calculations of both case counts and SEs for each category of procedure. We recommend using the fewest historical quarters, starting with the most recent quarter, either with at least 800 cases or an estimated asymptotic SE for the estimated percentage no greater than 1.25%. Applying our methodology with local LOS data will allow OR managers to estimate the number of patients on the elective OR schedule each day who will be hospitalized for longer than overnight, facilitating communication and decision-making with surgical departments when census considerations constrain the ability to run a full surgical schedule.

## Introduction

In scenarios where hospital census is high, perioperative medical directors or operating room (OR) managers may sometimes need to review with surgical departments as to which surgical cases scheduled to be performed within the next three days may need to be postponed, if clinically appropriate. This scenario has arisen at hospitals in regions with large increases in admissions due to coronavirus disease 2019 (COVID-19) and hospital administrative or governmental mandates to reserve available beds for such patients.

Previously, we examined summary measures for hospital length of stay (LOS), as could be used to guide the OR manager when deciding which cases may need to be postponed [1,2]. Distributions of LOS are highly skewed, complicating the decision-making process. We used data from all hospitals and ambulatory surgery centers in Florida to study all surgical cases that included at least one major therapeutic procedure, with procedures classified using Clinical Classifications Software (CCS) from the Agency for Healthcare Research and Quality (AHRQ) [3,4].

The first summary measure compared was the percentage of patients previously undergoing the same category of procedure as that scheduled whose hospital LOS was zero (i.e., ambulatory surgery) or one day (i.e., overnight stay) [1,2]. The percentage of cases with LOS of <2 days, being a single number, is simple to compute and easy to understand.

The second set of summary measures compared were the mean and standard deviation of LOS for each category of procedure [1,2]. Student’s t-test with unequal variances is robust to deviations from a normal or lognormal distribution [1]. However, for interpreting LOS, only considering the mean is insufficient because standard deviations differ markedly among procedures [1]. Consideration of both the mean and the standard deviation can be too challenging to be practical to assist decision-making related to case postponement.

The third measure compared was the probability that a patient undergoing one category of procedure would have a smaller LOS than a patient undergoing another category [1,2]. That probability is the area under the receiver operating characteristic curve, calculated by the Wilcoxon-Mann-Whitney test. Although this approach is distribution-free, it does not summarize each case as needed by the OR manager; rather, it provides pairwise comparisons among all cases [5,6].

The statistical power to compare cases of different categories of procedures was at least as large by comparing percentages of patients with LOS of <2 days than versus Student’s t-test with unequal variances or Wilcoxon-Mann-Whitney test [1]. We recommended applying the first measure because it is the easiest of the three to implement and interpret [1,2].

One application of our finding is for bed management during the COVID-19 pandemic when the hospital census is high and the number of beds available for elective admissions is being rationed [2]. The OR manager will have many issues in addition to LOS to consider with surgical departments when determining which cases to postpone, including, but not limited to, the patients’ medical conditions, their residential locations and travel times to the hospital, and surgeons’ availability. The implication of our results is that when balancing multiple competing objectives and making decisions for scores or hundreds of cases per week, the manager and surgeons can include in the deliberation of which cases to postpone the simple statistic of whether there is a high probability that the patient will remain in the hospital for two days or longer. Although the mode or median of the LOS for a given procedure may be known, this is insufficient to provide information about the tails of the distribution (i.e., the outliers) necessary for decision-making. For example, at the hospital studied, most patients undergoing nephrectomy had a LOS of two days (i.e., the mode was two days), and 54% of the patients had a LOS of ≤2 days (i.e., median was two days), but 31% had a LOS of ≥4 days. The OR manager contributes to the assurance of enough bed capacity for medical patients requiring admission (e.g., with respiratory failure from COVID-19).

In practice, applying our scientific results [1,2] would require that the hospital estimates for each category of procedure the percentage of cases that have LOS of <2 days. For example, the preceding 100 cases could be used for each category of procedure, and the percentage of those cases with LOS of <2 days calculated. This forecasting is a time series prediction problem. The advantage of using N = 100 is that the cases will be recent, thereby reflecting current discharge practices. Alternatively, the last 800 cases could be used. The advantage of using N = 800 is that the percentage of the cases with LOS of <2 days will be estimated more accurately. However, such thresholds are arbitrary. Alternatively, for each category of procedure, the smallest number of preceding cases possible could be used for which the standard error (SE) of each percentage estimate is less than some threshold (e.g., 1.25%). The advantage of an approach based on the SE is that to estimate the percentage of cases with LOS of <2 days, many fewer cases are needed to estimate the percentage within approximately 2.5% when the raw percentage is approximately 95% than when the raw percentage is close to 50%. A further advantage is that consideration of the SE balances the desire to use the most recent cases (i.e., favoring current practice), and the desire to obtain greater precision (i.e., larger sample size). In this study, we analyzed five years of data from a large teaching hospital to compare methods of estimating the percentages of cases with LOS of <2 days for each category of procedures.

## Materials and methods

The University of Iowa Institutional Review Board determined on September 2, 2020, that this project (No. 202008508) does not meet the regulatory definition of human subjects research since it involved the analysis of previously collected and de-identified data. Table 1 shows the progressive process of selecting cases for the training and testing datasets.

**Table 1:** Progressive selection of cases studied ^a^We could have created periods from June 30, 2020, backward rather than from July 1, 2019, forward. There would still have been 13 four-week periods. The consequence would have been that with the exclusion of slowdown days from the pandemic, there would have been two four-week periods, each with only five workdays. We made the more conservative choice of having 12 instead of 13 four-week periods (i.e., one less degree of freedom) so that each period had more workdays. The conservative choice means that we ensured greater reliability of our results CCS: Clinical Classifications Software

Cases	Progressive selection of cases
147,104	Cases performed in the main hospital surgical suites, pediatric hospital, and ambulatory surgery center, from Thursday, October 1, 2015, through Tuesday, June 30, 2020, 19 consecutive quarters over a five-year period. The pediatric cases were shifted from the main hospital to a new hospital building in April 2017
124,719	Excluded 22,385 urgent and emergent cases
124,469	Excluded 250 cases with no matching CCS category of procedure (e.g., excludes patients brought to the operating room solely for tracheal intubation)
124,469	Cases available for assignment
98,540	Assigned to the training dataset (October 1, 2015, through June 30, 2019)
25,929	Provisionally assigned to the test dataset (July 1, 2019, through June 30, 2020)
25,703	Excluded 226 cases performed during the period June 28-30, 2020, because Monday, July 1, 2019, through Sunday, June 27, 2020, represented precisely 13 four-week periods^a^
24,657	Excluded 1,046 cases performed during the slowdown period at the start of the COVID-19 pandemic in Iowa. Every non-weekend date, October 1, 2015, through June 30, 2020, with at least five cases but less than 50 cases were performed during this period
24,338	Excluded 319 cases performed during the 11th of the 13 four-week periods, because the 11th period now only contained four successive workdays, at the end of the slowdown period^a^
24,338	Assigned to the testing dataset (July 1, 2019, through June 27, 2020)

The surgical cases were performed in the main adult surgical suites, the ambulatory surgery center, or the pediatric suite ORs at the studied hospital, all located within a five-minute indoor walk of one another [7]. The cases were limited to those that were elective, defined by the organization as the surgeon having specified that the patient could wait safely at least three days for surgery (i.e., from Friday to Monday). The start date, October 1, 2015, was selected because that was the change date in the United States when all inpatient procedures were required to be coded using the International Classification of Diseases Version 10 Procedure Coding System (ICD‑10-PCS). We therefore only needed to use the CCS categories obtained from ICD-10-PCS and for Current Procedural Terminology (CPT) codes [3,4]. The last date of cases was June 30, 2020, the end of the last full quarter in 2020 before data analysis. LOS was estimated with discharge data through August 2020. There were 12 four-week periods available for testing during the final year. Table 2 shows that the distribution of included cases among surgical specialties was similar between the two periods.

**Table 2:** Distribution of surgical specialties among included cases, listed for the specialties each with at least 1% of all cases ^a^There were six additional specialties, not listed because each accounted for less than 0.6% of the studied cases ^b^The sample sizes are explained in Table 1

Specialty^a^	Historical period (n = 98,540 cases)^b^	Test period (n = 24,338 cases)^b^
Orthopedics	25.27%	28.39%
General surgery adult and plastics	14.71%	14.72%
Ophthalmology	12.52%	11.82%
Otolaryngology	12.20%	11.06%
Neurosurgery	7.83%	7.13%
Gynecology	7.18%	8.04%
Urology	6.10%	6.34%
Cardiothoracic	3.85%	3.22%
Dentistry and oral surgery	3.16%	2.89%
Pediatrics	2.71%	2.24%
Vascular	2.50%	2.24%
Neuroradiology	1.05%	0.96%

For assigning a CCS category to each case, the vast majority of cases had only one procedure, or when more than one procedure was performed during the case, all procedures performed were of the same category. When a case included more than one procedure and the procedures mapped to more than one CCS category, we used data from the State of Florida [2]. For each CCS category of major therapeutic procedures, we have previously used the percentage of patients in Florida in 2018 who had LOS of <2 days [2]. The CCS assigned to the case at the study hospital was the CCS with the smallest percentage of patients with LOS of <2 days [2]. The 10 most common of the 185 observed CCS categories are listed in Table 3.

**Table 3:** Distribution of Clinical Classifications Software (CCS) categories of procedures among included cases, listed for the CCS categories, each with at least 2% of all cases ^a^In addition to the 10 above-listed CCS categories of procedures, there were 175 other categories, each with at least one case during the historical or test periods. Among those 175, there were 26 that each accounted for greater than 1.0% but fewer than 2.0% of the studied cases ^b^The sample sizes are explained in Table 1

CCS^a^	Historical period (n = 98,540 cases)^b^	Test period (n = 24,338 cases)^b^	CCS label
161	4.75%	3.78%	Other operating room therapeutic procedures on bone
160	4.37%	4.72%	Other therapeutic procedures on muscles and tendons
15	4.22%	4.11%	Lens and cataract procedures
170	3.66%	3.13%	Excision of skin lesion
162	2.82%	3.02%	Other operating room therapeutic procedures on joints
23	2.62%	2.16%	Myringotomy
29	2.49%	2.11%	Oral and dental services
6	2.06%	3.72%	Decompression peripheral nerve
67	2.34%	2.26%	Other therapeutic procedures, hemic and lymphatic system
175	2.32%	2.05%	Other operating room therapeutic procedures on skin and breast

Training dataset segmented by quarters

All estimation for cases performed during each quarter corresponding to the test dataset was done based on the LOS percentages from cases performed before the start of that quarter (Table 1). We applied this approach because we expect that many hospitals would not update computer tables more frequently than on a quarterly basis. For example, estimates of the percentage of cases with LOS of <2 days for all cases in the 19th quarter were forecast using workdays in one or more of the preceding 18 quarters. From each quarter, the data used were the count of elective cases for each of the categories and the count of such cases with LOS of <2 days. Estimation for each quarter proceeded as described below.

For each category of procedure, we calculated the cumulative count of cases among quarters, from the most recent quarter, second most recent quarter, and so forth up to the quarter resulting in at least 800 cases. If all preceding quarters combined had fewer than 800 cases, we included all previous cases. Among the 185 observed CCS categories of procedure, there were 133 with fewer than 800 cases in total. The choice of 800 cases was based on the results as described in the below paragraphs. For each combination of category and quarter, we used the cumulative counts of cases and cases with LOS of <2 days, excluding the current quarter.

For each category of procedures, and for each of the preceding quarters included for the category, we used the cumulative counts to calculate the asymptotic SE for the proportion of cases with LOS of <2 days. That equaled the square root of three terms: the proportion multiplied by the quantity one minus the proportion then divided by the count of cases among the included preceding quarters. If all preceding quarters combined provided a sample size such that the estimated SE for the proportion exceeded 1.25%, we included the cases from all preceding quarters.

For each combination of the category of procedure and quarter, we determined the smallest number of preceding quarters needed to be included for the preceding count of cases to be at least 800. We also determined the smallest number of preceding quarters needed to be included for the estimated SE of the proportion to be ≤1.25%. We used whichever of the two that permitted the use of fewer preceding quarters of data.

Applying the training dataset results to the test dataset

As noted in the legend of Table 1, there were N = 12 included four-week periods during the final year, comprising the test dataset. For each of the four-week periods, we calculated the count of elective cases, the count of elective cases for each of the categories of procedures, as well as the count of such cases with LOS of <2 days for each of the categories. Applying the training dataset as described in the preceding section, we also had for each case performed during the training dataset a forecasted probability that the case would have LOS of <2 days. We summed the forecasted probabilities to give an estimate for the count of cases with LOS of <2 days during the period. We summed the forecasted probabilities because the expected value of the sum of independent Bernoulli trials equals the sum of the individual probabilities.

The quality of the estimates was calculated for each four-week period. For each period, the observed percentage of cases with LOS of <2 days was subtracted from the estimated percentage of cases with LOS of <2 days. The absolute value of the difference was taken. The sample mean and sample SE of the mean of the absolute values were calculated among the periods. All statistical inference was performed using two-sided one-group Student’s t-tests based on the sample size of N = 12 independent four-week periods. Data are reported as mean (SE) except for data from Florida [2], which were reported as percentages and SE.

## Results

The studied hospital averaged 112 [1] elective cases per workday. Among all hospitals and ambulatory surgery centers in Florida, 72.88% (0.03%) of cases had LOS of zero or one day [2]. At the studied hospital, the percentage was 73.1% (0.4%) (i.e., no different from that in Florida, P = 0.53). Thus, we believe these findings can be generalized, even though the data in this study are from a single large teaching hospital.

We varied the threshold sample size in increments of 25 historical cases and the threshold SE of the percentage of cases with LOS of zero or one day in increments of 0.25%. The minimum observed absolute percentage error was 0.76% (0.12%), achieved by using the minimum number of quarters of the year of data providing for either a minimum of 800 historical cases or a maximum 1.25% SE (see Materials & Methods). Table 4 shows that there were multiple, near-equivalent, and suitable choices for optimal parameter choices.

**Table 4:** Quality of forecasted probabilities of cases having a length of stay (LOS) of <2 days The means are reported with the standard errors (SEs) of the means among the test period of N = 12 four-week periods (see Table 1). The parameter values described in the section Materials & Methods and used in Table 4 are noted with an asterisk. The negative numbers for bias in the third column show that the historical data (i.e., from the training period) underestimated the percentages of cases with LOS <2 days during the test period. This indicates that, overall, the studied hospital's LOS was declining progressively over time

Preceding quarter’s minimum sample size	Preceding quarter’s maximum achieved SE of the percentage cases with LOS of <2 days	Mean (SE) bias in the estimated percentage	Mean (SE) absolute difference in the estimate's percentage
700	1.25%	-0.079% (0.270%)	0.800% (0.125%)
750	1.25%	-0.112% (0.260%)	0.756% (0.129%)
800	1.25%	-0.130% (0.256%)	0.755% (0.123%)
850	1.25%	-0.158% (0.255%)	0.758% (0.124%)
900	1.25%	-0.177% (0.259%)	0.768% (0.128%)
800	0.75%	-0.246% (0.257%)	0.777% (0.128%)
800	1.00%	-0.190% (0.255%)	0.765% (0.122%)
800	1.25%	-0.130% (0.256%)	0.755% (0.123%)
800	1.50%	-0.115% (0.259%)	0.766% (0.122%)
800	1.75%	-0.026% (0.280%)	0.827% (0.128%)
750	1.00%	-0.168% (0.256%)	0.761% (0.125%)
750	1.50%	-0.088% (0.263%)	0.767% (0.129%)
850	1.00%	-0.215% (0.253%)	0.766% (0.123%)
850	1.50%	-0.138% (0.257%)	0.763% (0.123%)

There were 167 CCS categories of procedure with at least one case among the test periods. For each category, we calculated among all periods the absolute difference between the estimated and the actual count of cases with LOS of zero or one day. We calculated the contribution of each category to the mean. Table 5 lists the categories with the largest errors.

**Table 5:** Clinical Classifications Software (CCS) categories of procedures with the least accurate predictions of length of stay (LOS) of <2 days Among the 167 CCS categories, the nine listed were the least accurate, with each contributing at least 2% to the overall mean 0.0.13% absolute error in the forecast percentages of cases with LOS of <2 days. Among the 158 categories not shown, there were 21 accounting for at least 1.0% but less than 2.0% of the mean absolute error. The sum of the percentage contributions to the mean absolute error among all 167 CCS categories was 100% OR: operating room

Percentage contribution	% LOS	Mean estimated % LOS of <2 days	% total case count	CCS	CCS Label
9.46%	60%	67%	3.7%	6	Decompression peripheral nerve
5.57%	56%	71%	1.1%	148	Other fracture and dislocation procedure
4.74%	70%	63%	1.9%	152	Arthroplasty knee
4.31%	45%	35%	1.3%	3	Laminectomy, excision intervertebral disc
3.91%	73%	70%	3.8%	161	Other OR therapeutic procedures on bone
3.58%	69%	62%	1.5%	9	Other OR therapeutic nervous system procedures
3.26%	82%	77%	2.0%	175	Other OR therapeutic procedures on skin and breast
3.24%	88%	95%	1.4%	149	Arthroscopy
2.29%	22%	26%	1.8%	61	Other OR procedures on vessels other than head and neck

## Discussion

Our primary result was that an average absolute percentage error of only 0.76% can be achieved for the percentage probability of patients having LOS of <2 days. This error is nearly 100-fold smaller than the percentage of cases to which it would be used (i.e., 0.76% versus 73.1%). Furthermore, Tables 4, 5 show that most of this small bias was caused by progressive reductions in the overall LOS over time (i.e., there was a consistent underestimation of the percentages of cases with LOS of <2 days). For the application of predicting cases' LOS when the census is high, such underestimation would be preferred because it is conservative. In other words, the number of patients staying in the hospital for ≥2 days would be slightly less than predicted.

One limitation of our study is that we used data from one large teaching hospital. However, there was no difference in its percentage LOS of <2 days than among all hospitals and ambulatory surgery centers in Florida. Thus, we believe these findings can be generalized.

A second limitation is that we used two criteria for selecting historical quarters of training data for each category of procedure, while there are other potential predictive models (e.g., linear changes in percentages over time). However, we doubt that this is important. Table 4 shows that there were multiple, nearly equivalent choices for the optimal parameter choices. What matters is that our conceptual strategy worked well, with the precise value of the parameters being of minimal importance.

## Conclusions

When estimating percentages of cases with LOS of <2 days, a decision needs to be made as to how much historical data to use. Including too many cases would increase the predictive error because of reductions in LOS over time for many procedures. Including too few cases would increase the predictive error because of imprecision (i.e., wide prediction intervals). In this study, we show that when performing these time series calculations quarterly, a reasonable approach is to perform calculations of both case counts and SEs for each category of procedure. We recommend using the fewest historical quarters, starting with the most recent quarter, either with at least 800 cases or an estimated asymptotic SE for the estimated percentage no greater than 1.25%. The application of our methodology with local LOS data will enable OR managers to estimate the number of patients on the elective OR schedule each day who will be hospitalized for longer than overnight, thereby facilitating communication and decision-making with surgical departments when census considerations hamper the ability to run a full surgical schedule.
